# Significantly Enhanced Corona Resistance of Epoxy Composite by Incorporation with Functionalized Graphene Oxide

**DOI:** 10.3390/ma17194864

**Published:** 2024-10-02

**Authors:** Yue Yang, Yumin Wang, Chunqing He, Zheng Wang, Xiangyang Peng, Pengfei Fang

**Affiliations:** 1 School of Physics and Technology, Wuhan University, Wuhan 430072, China; yangyue@whu.edu.cn (Y.Y.); 2015301020065@whu.edu.cn (Y.W.); hecq@whu.edu.cn (C.H.); 2 Guangdong Key Laboratory of Electric Power Equipment Reliability, Electric Power Research Institute of Guangdong Power Grid Co., Ltd., Guangzhou 510080, China; williamatwhu@163.com (Z.W.); pigpxy@126.com (X.P.)

**Keywords:** epoxy resin, graphene oxide nanosheets, corona discharge resistance, electrochemical impedance spectroscopy

## Abstract

Enhancing the corona resistance of epoxy resin (EP) is crucial for ensuring the reliable operation of electrical equipment and power systems, and the incorporation of inorganic nanofillers into epoxy resin has shown significant potential in achieving this. In this study, functionalized graphene oxide (KHGO) was synthesized via a sol-gel method to enhance the corona resistance of EP with electrochemical impedance spectroscopy (EIS) used to assess the properties of KHGO/EP composites. Fourier transform infrared spectroscopy (FTIR) and X-ray photoelectron spectroscopy (XPS) verified the successful grafting of epoxy groups onto the GO surface. The thermal conductivity and stability of the KHGO/EP composite initially increased with KHGO content but declined when the content exceeded 1.2 wt.%. Positron annihilation lifetime spectroscopy (PALS) indicated that KHGO improved interfacial compatibility with EP compared to GO, with agglomeration occurring when KHGO content exceeded a threshold value (1.2 wt.%). EIS analysis revealed that the corona resistance of the KHGO/EP composite was optimal at a filler content of 0.9 wt.%. After corona treatment, the saturation water uptake of the 0.9 wt.% KHGO/EP composite decreased by 15% compared to pure EP with its porosity reduced to just 1/40th of that of pure EP. This study underscores that well-dispersed KHGO/EP composite exhibits excellent corona resistance property suggesting the potential for industrial applications in high-voltage equipment insulation.

## 1. Introduction

Epoxy resin (EP) materials are extensively utilized in power systems due to their outstanding insulation and mechanical properties, making them indispensable in various electrical equipment applications [1,2]. However, during operation, epoxy resins are susceptible to corona discharge under the combined influence of strong electric fields and environmental factors [3]. Exposure to corona discharge can lead to degradation and cracking of the epoxy resin, ultimately resulting in insulation failure and compromising the operational safety of the equipment [4,5]. Investigating the aging process and mechanisms of epoxy resin under corona discharge is crucial. Identifying strategies to enhance its corona resistance is of significant practical importance for advancing the applications of epoxy resin in high-voltage insulation [6].

In existing research, there are two main methods to enhance the corona resistance of polymers: surface modification [7] and the addition of inorganic nanofillers [8,9,10,11]. Among these, studies on surface modification are relatively few, while research on nanofillers dominates [12,13,14]. For instance, An et al. have treated clean epoxy resin with a mixture of fluorine and nitrogen gases to form a micron-thick, fluorinated layer on the surface of epoxy [15,16]. Experiments demonstrated that the creation of this fluorinated layer enhanced the rate of surface charge decay, effectively reducing the accumulation of surface charges and thereby improving the corona resistance of the epoxy resin matrix. At present, a range of inorganic nanofillers is utilized to improve the corona resistance of polymeric materials [17,18]. These nanofillers can be categorized by types of substances, including aluminum oxide, aluminum hydroxide, silicon dioxide, silicates, titanium dioxide, zinc oxide, and magnesium oxide, among others [19,20,21]. Among these, nanoparticles of aluminum oxide and silicon dioxide are widely available, cost-effective, and have the potential for industrial-scale production. These nanofillers can also be classified by morphology into nanoparticles, nanotubes, and nanosheets [22,23,24]. Due to the relatively simple preparation process of nanoparticles, past research has predominantly focused on nanoparticles. Additionally, some researchers have investigated the synergistic effects of different types of inorganic fillers in enhancing the corona resistance of polymer materials [25]. Inorganic fillers possess stable physical and chemical properties, making them resistant to decomposition under corona discharge, thereby providing a physical barrier effect [26,27,28]. Because of the high specific surface area of nanoparticles, they can form extensive interfacial layers with the polymer matrix, thereby acting as channels for the transport of space charges within the polymer [29,30]. In summary, nanoparticles enhance the corona resistance of polymer materials by providing physical barriers, improving thermal conductivity, and suppressing space charge accumulation.

Nowadays, nanosheet fillers have attracted interest in the development of corona-resistant polymer composites, with materials such as silicate nanosheets and boron nitride nanosheets [31,32,33]. Nanosheets not only improve the thermal conductivity of polymer materials more efficiently but their large aspect ratio also provides excellent physical barrier properties [34]. Theoretically, nanosheets are superior to nanoparticles as corona-resistant fillers. Graphene oxide (GO) features a surface rich in hydroxyl, carboxyl, and epoxy groups, giving it high chemical reactivity [35]. The abundant active groups on the GO surface not only facilitate silane modification but also improve its dispersibility in organic matrices [36]. More importantly, graphene exhibits very high electrical conductivity, which is detrimental for applications in insulating materials. In contrast, highly oxidized graphene oxide (GO) has low electrical conductivity, making it a promising nanoscale insulating filler [37].

In this article, an epoxy-functional silane coupling agent (KH560) was used for surface modification of GO via a sol-gel method, aiming to enhance its dispersion within the epoxy resin matrix. EP composites containing varying amounts of modified GO (KHGO) content were synthesized. Positron annihilation lifetime spectroscopy technology was utilized to investigate the dispersion of KHGO in the EP matrix. Due to the disordered atomic structure of polymer composites, characterizing their structure is very difficult. Thus, in previous studies, there have primarily been two methods for characterizing the corona resistance of polymers: surface roughness analysis and breakdown life testing. This study is the first to apply electrochemical impedance spectroscopy (EIS) to the evaluation of corona resistance in polymer materials, demonstrating the advantages of EIS in characterizing the internal structure of polymer materials.

## 2. Materials and Methods

### 2.1. Sample Preparation

#### 2.1.1. Surface Modification of GO

The surface modification mechanism of GO is shown in Figure S1 [38,39,40]. The monolayer or few-layer GO nanosheets used in this article are produced by Hummer’s method [41]. First, a mixed solution of 100 mL anhydrous ethanol, water, and silane coupling agent KH560 was prepared in a volume ratio of 75:10:15. Then, 1 g of graphene oxide was dispersed in the mixed solution by ultrasonication. Subsequently, the pH of the suspension was fine-tuned to a range of 4.2 to 4.5 by adding glacial acetic acid dropwise. Finally, the mixture was allowed to stand for 24 h. After the aforementioned process was completed, while stirring continuously, a dropwise addition of 1 mol/L NaOH solution was made to the suspension to raise the pH to 8.5. The suspension was subsequently heated in a water bath at 60 °C for 1 h. After heat treatment, the suspension was centrifuged at high speed. The resulting precipitate was washed four times with a 1:1 mixture of anhydrous ethanol and water by volume, followed by one additional wash with anhydrous ethanol. Finally, the washed precipitate was dried to obtain KHGO powder.

#### 2.1.2. Preparation of GO/EP Composites

In this article, the di-glycidyl ether of bisphenol-A (DGEBA) epoxy resin was used. Initially, 5 g of DGEBA was dissolved in a mixed solvent consisting of 1.6 g of xylene and 1 g of n-butanol. The solution was stirred with a clean glass rod until the DGEBA was fully dissolved. Next, a measured amount of KHGO powder was incorporated into the solvent mixture. The beaker was subsequently sealed and set on a magnetic stirrer for 24 h. After stirring, 2.5 g of a modified aliphatic amine epoxy hardener was introduced, and the mixture was agitated for 5 min to ensure thorough dispersion. To remove dissolved air, the mixture underwent ultrasonic treatment for 5 min. The resulting solution was spin-coated onto the conductive surface of clean ITO glass, with the spin-coating parameters adjusted to achieve a coating thickness of 30 ± 2 μm. The samples were cured in an electric blast drying oven at 40 °C for 24 h, followed by a postcuring process at room temperature for over 7 days. Additionally, square and disc-shaped samples were prepared using polytetrafluoroethylene (PTFE) molds to evaluate the thermal and electrical properties of the composites.

### 2.2. Corona Discharge Treatment

A schematic diagram of the homemade corona discharge apparatus is shown in Figure S2. The electrodes, both needle and plate types, were manufactured using stainless steel. The needle electrode featured a diameter of 3 mm and a curvature radius of 50 μm, whereas the plate electrode had a diameter of 20 mm and a thickness of 5 mm. To prevent short circuits, a silicone rubber insulating gasket with a thickness of 1 mm was placed between the sample and the plate electrode, covering the latter. Since corona discharge is sensitive to environmental humidity, all experiments were performed in conditions where the relative humidity (RH) varied between 30% and 65%. The high-voltage power supply used was a dielectric strength tester with a maximum output voltage of 20 kV. In this article, EIS measurements for all samples were made after 6 kV corona discharge treatment for 3 h.

### 2.3. Characterization

The micromorphologies of GO and KHGO nanosheets were examined using scanning electron microscopy (SEM, Hitachi S-4800, Hitachi, Chiyoda, Japan), transmission electron microscopy (TEM, JEOL JEM-2100 F, JEOL Ltd., Tokyo, Japan), and atomic force microscopy (AFM, Bruker Dimension Icon XR, Bruker, Billerica, MA, USA). The chemical structure of GO and KHGO were analyzed by Fourier transform infrared spectroscopy (FTIR, Thermo Fisher Nicolet 5700, Thermo Fisher Scientific, Waltham, MA, USA) with a resolution of 4 cm^−1^, X-ray diffraction (XRD, Bruker D8-advance) with a scan rate of 5°/min and X-ray photoelectron spectroscopy (XPS, Thermo Fisher ESCALAB 250Xi). The thermal diffusivity of composite samples was measured by laser flash apparatus (Bruker, LFA-467). TG curves of composite samples were measured by a simultaneous thermal analyzer (Bruker, STA-449C) at a heating rate of 10 K/min from 30 °C to 600 °C. The volume conductivity of composite samples was measured using a custom-built laboratory platform. The testing platform utilized a dielectric strength tester as the high-voltage power source and two copper plates as electrodes. During testing, the square-shaped sample was placed between the copper electrodes, and voltage was applied. A microammeter was used to measure the current flowing through the sample.

The positron annihilation lifetime was measured using a fast–fast coincidence lifetime spectrometer (ORTEC, Atlanta, GA, USA). The positron source used was ^22^Na encapsulated with Kapton film, with a measurement precision of 300 ps. The thickness of the test samples was 1.5 mm. A sandwich structure with the positron source placed between two sample pieces was employed for the measurements, and each sample was subjected to a total count of one million. Before testing the composite samples, the positron lifetime spectrum of single-crystal Ni was measured to derive the annihilation information of the positron source in the Kapton film, which is referred to as source correction. The test data were analyzed and fitted using the PATFIT software (version 9.0).

The electrochemical impedance spectroscopy (EIS) measurements were taken by an electrochemical workstation (Corrtest Instruments, CS310, Wuhan Corrtest Instruments Corp., Ltd., Wuhan, China). The EIS spectra of the samples treated with corona discharge were measured using a three-electrode system in this article. The working electrode used was ITO, with platinum as the counter electrode and a saturated calomel electrode (SCE) as the reference. The system was enclosed within a Faraday cage for testing, with a perturbation voltage fixed at 20 mV and a frequency range of 10^5^–10^−2^ Hz.

## 3. Results and Discussion

### 3.1. Chemical Structure and Morphology of Nanosheets

The hydroxyl and carboxyl groups present on the surface of GO facilitate its dispersion within the organic EP matrix. In this study, KH560 was employed to functionalize the surface of GO with the aim of introducing epoxy groups onto its surface. During the curing process of the composite, the epoxy groups on the surface of KHGO can crosslink with the epoxy groups on the epoxy molecular chains, thereby enhancing the interface between KHGO and the epoxy matrix and further improving its dispersion. To investigate the results of surface modification on the chemical structure of GO, this study first conducted XRD, FTIR, and XPS analyses on both GO and surface-modified KHGO.

**Figure 1:**
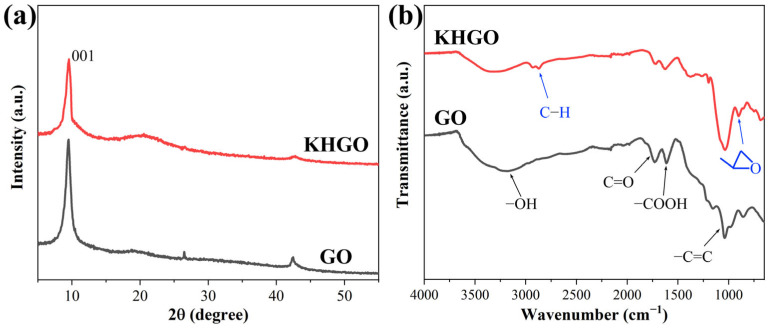
XRD spectra (**a**) and FTIR spectra (**b**) of GO and KHGO.

Figure 1a shows the XRD patterns of both GO and KHGO. The diffraction peak of GO appears at 2θ = 9.7°, corresponding to the (001) plane of graphene oxide [42]. Similarly, the diffraction peak of KHGO appears at 2θ = 9.5°, also corresponding to the (001) plane of graphene oxide. The XRD results indicate that the lattice structure of GO did not undergo significant changes during the sol-gel process used to prepare KHGO. Literature reports that surface-modified graphene oxide typically exhibits an increased interlayer spacing, causing a leftward shift of the (001) diffraction peak [43]. The experimental results of this study show a very slight leftward shift of the (001) diffraction peak in KHGO compared to GO.

Figure 1b illustrates the FTIR spectra of GO and KHGO. The FTIR spectrum of GO exhibits a peak at 3300 cm^−1^ representing hydroxyl groups, two peaks near 1700 cm^−1^ representing carbonyl and carboxyl groups, and a peak at 1100 cm^−1^ representing carbon-carbon bonds in graphene oxide [44]. In the KHGO FTIR spectrum, two new absorption peaks appear at 2900 cm^−1^, along with an additional peak at 900 cm^−1^. The doublet or triplet peaks around 2900 cm^−1^ are typically associated with C-H stretching vibrations in methyl groups, indicating the presence of methyl groups on the KHGO surface [45]. The absorption peak at 900 cm^−1^ is characteristic of epoxy groups, suggesting that the epoxy groups in KH560 molecules were successfully attached to the GO surface.

**Figure 2:**
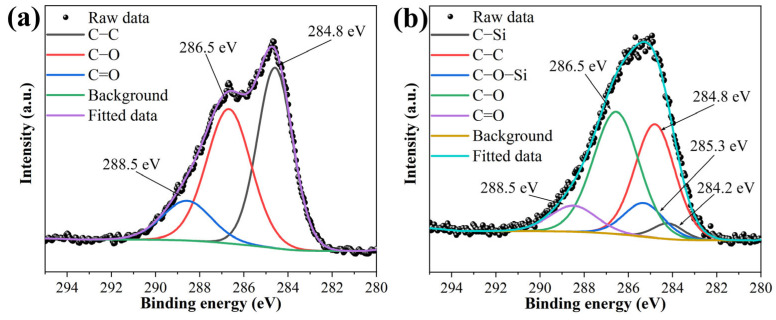
High-resolution XPS spectra of C 1s in GO (**a**) and KHGO (**b**).

The XPS overview spectra for GO and KHGO are presented in Figure S3. In the XPS survey spectrum of GO, there are two peaks corresponding to O 1s and C 1s [46]. In the XPS survey spectrum of KHGO, along with the O 1s and C 1s peaks, a Si 2p peak is also observed. This result corroborates the findings from the FTIR results. The deconvolution of the C 1s peaks for GO and KHGO are shown in Figure 2a,b, respectively. In the high-resolution XPS spectrum of the C 1s peak in GO, two distinct peaks are observed, whereas in KHGO, the C 1s peak appears as a single envelope. For GO, the C 1s spectrum indicates three primary carbon chemical states: C–C, C=O, and COOH, with corresponding binding energies of 284.8 eV, 286.5 eV, and 288.5 eV [47]. In KHGO, the chemical states of carbon are more complex, including the three states found in GO as well as new states introduced by the chemical reaction, such as C–Si and C–O–Si, with binding energies of 284.2 eV and 285.3 eV, respectively. The XPS results further confirm that the epoxy groups from KH560 were successfully introduced onto the surface of the graphene oxide.

**Figure 3:**
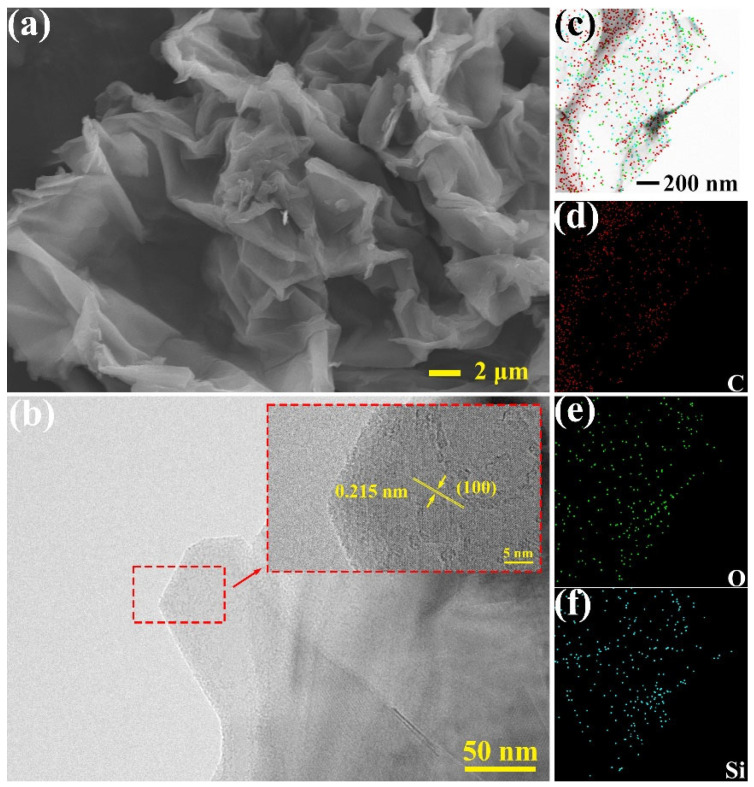
SEM image (**a**) and TEM image (**b**) of GO; elemental distribution on the surface of GO (**c**–**f**).

The SEM image of unmodified GO is shown in Figure S4a. At the micron scale, the two-dimensional sheet structure of graphene can be observed. The surface wrinkles are due to the thin and flexible nature of GO, which contracts under van der Waals forces. The TEM image of GO is shown in Figure S4b, where extremely thin GO layers can be seen supported on a mesh-like carbon grid. Elemental scanning analysis using EDS was performed on the GO surface, resulting in elemental maps of C, O, and Si, as shown in Figure S3c–f. From the EDS maps, it can be seen that the red dots representing C and the green dots representing O are densely distributed throughout the region, whereas the blue dots representing Si are very sparsely distributed. The elemental composition in the test area was calculated as follows: 76.58% C, 23.40% O, and 0.02% Si, the latter being negligible. The SEM image of surface-modified KHGO is shown in Figure 3a. Similar to GO, KHGO exhibits a two-dimensional sheet structure with numerous wrinkles formed due to van der Waals forces acting on the thin layers. The TEM image of KHGO is presented in Figure 3b. Lattice fringes were observed at the edges of KHGO, with an interlayer spacing measured at 0.215 nm, representing the (100) plane of graphene oxide [48]. The SEM and TEM results indicate that the thin two-dimensional sheet structure of GO was maintained during the surface modification process with KH560. Elemental scanning analysis using EDS on the KHGO surface yielded elemental maps for C, O, and Si, as shown in Figure 3c–f. From the EDS maps, it is evident that the distribution range and density of the blue dots representing Si significantly increased. The elemental composition of KHGO was found to be 52.38% C, 37.01% O, and 9.71% Si. Compared to GO, the proportion of Si in KHGO increased substantially, and the proportion of O also increased. As shown in Figure S1, the functional groups of KH560 were connected onto the surface of GO during the modification process, which introduced the Si element and O element. These results further confirm that the functional groups of the KH560 molecules successfully attached to the GO surface.

**Figure 4:**
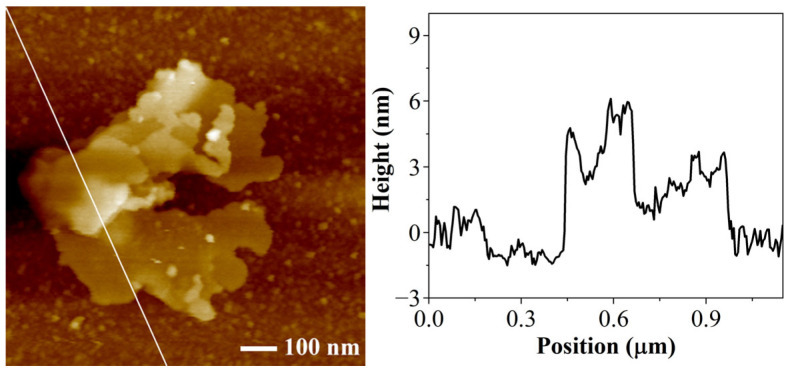
AFM image of KHGO and thickness distribution along the white line.

The AFM image and line scan results of KHGO are shown in Figure 4. The AFM image reveals that KHGO exhibits a two-dimensional sheet structure, with overlapping sheets observed within the scanned area. In the line scan results, the intervals with a height of 0 represent the silicon wafer surface, while other intervals represent the sample surface. It is observed that the thickness in the overlapping regions of the sheets reaches 6 nm, whereas the thickness in the nonoverlapping regions is approximately 3 nm. Furthermore, the AFM image indicates that the lateral dimensions of the KHGO sheets are nearly 1 μm, suggesting an aspect ratio close to 300. The AFM results further confirm that the KHGO obtained through the sol-gel process maintains a very thin thickness and a very high aspect ratio.

### 3.2. Thermal Properties and Volume Conductivity of Composites Samples

**Figure 5:**
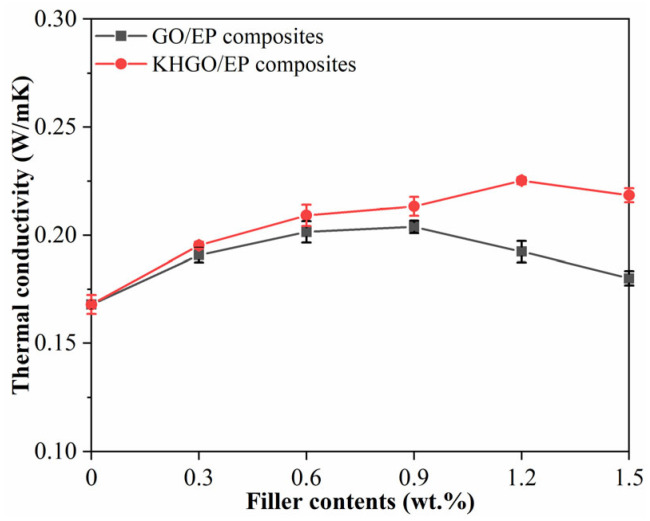
Thermal conductivity of GO/EP composites and KHGO/EP composites with different filler content.

In this article, the thermal diffusivity of EP composites with varying content of GO and KHGO was measured at room temperature using a laser flash apparatus. As depicted in Figure 5, the thermal conductivity was calculated based on the thermal diffusivity values [49]. Analysis reveals that the thermal conductivity of pure epoxy resin is approximately 0.17 W/mK. For the GO/EP composites, the thermal conductivity rises with increasing GO content, reaching a maximum value (approximately 0.20 W/mK) at a GO content of 0.9 wt.%. Beyond this concentration, the thermal conductivity decreases. Similarly, for the KHGO/EP composites, the thermal conductivity increases with the KHGO content, reaching a maximum value of approximately 0.23 W/mK at a KHGO content of 1.2 wt.%. Additionally, at the same filler content, the thermal conductivity of the KHGO/EP composites is slightly higher than that of the GO/EP composites. When GO or KHGO is incorporated into the epoxy resin matrix, two main factors contribute to the enhanced thermal conductivity of the composites. Firstly, the intrinsic thermal conductivity of the two-dimensional layers is higher than that of the epoxy resin matrix. Secondly, an interfacial layer forms between the epoxy resin matrix and the incorporated inorganic fillers. According to the existing interfacial layer theory in nanodielectrics, the interfacial layer, which has a certain thickness, forms between the polymer matrix and the inorganic fillers. The molecular structure within the interfacial layer differs from that of the polymer matrix. Generally, influenced by the regularly arranged atomic structure of the inorganic fillers, the molecular arrangement within the interfacial layer is more ordered than that within the polymer matrix. This enhanced molecular order typically leads to better crystallinity and, consequently, better thermal conductivity. Due to the combined effect of these two factors, epoxy resin composites containing GO or KHGO exhibit higher thermal conductivity than pure epoxy resin. As the filler content increases, the thermal conductivity improves. However, the observed phenomenon of thermal conductivity increasing initially and then decreasing with a further increase in filler content is likely related to the agglomeration of the two-dimensional layers. Given the exceptionally high specific surface area of the two-dimensional layers, they tend to agglomerate under van der Waals forces during the curing process of the epoxy resin composites. When agglomeration occurs, it diminishes the effectiveness of both factors that enhance the thermal conductivity of the polymer matrix. Consequently, at high filler content, the thermal conductivity of the composites decreases.

**Figure 6:**
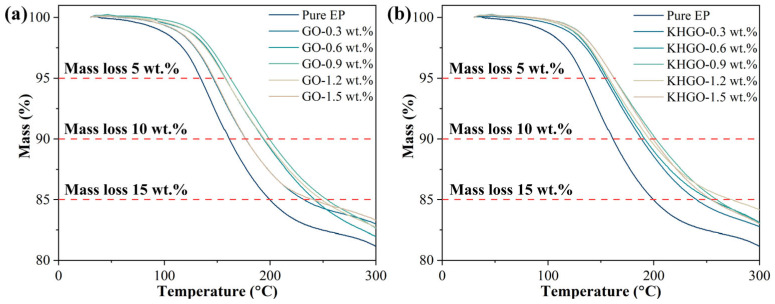
TG curves of GO/EP composites (**a**) and KHGO/EP composites (**b**) with different filler content from 30 °C to 300 °C.

The TG curves of epoxy resin composites with varying content of GO and KHGO, measured from 30 °C to 600 °C, are shown in Figure S5a,b. In order to more clearly differentiate the TG curves of the various samples, the test results within the temperature range from 30 °C to 300 °C were plotted, as illustrated in Figure 6a,b, respectively. It can be observed from these figures that the TG curves of epoxy resin composites containing inorganic fillers shift towards higher temperatures compared to the TG curve of pure epoxy resin. This shift indicates that the thermal stability of the composites is superior to that of pure epoxy resin.

**Figure 7:**
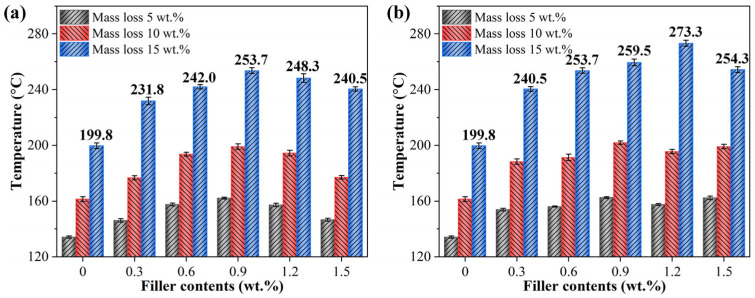
The temperature in TG tests when the weight loss of GO/EP composite (**a**) and KHGO/EP composites (**b**) reach 5 wt.%, 10 wt.% and 15 wt.%.

To provide a more intuitive analysis of the thermal stability of epoxy resin composites with different filler content, bar charts of the temperatures at which the mass loss in the TG curves reaches 5 wt.%, 10 wt.%, and 15 wt.% are shown in Figure 7a,b. The bar chart results indicate that, with increasing GO or KHGO content, the thermal stability of the epoxy resin composites initially increases and then decreases, exhibiting a trend similar to that of thermal conductivity. Similarly, at the same filler content, the thermal stability of KHGO/EP composites is better than that of GO/EP composites. The phenomenon of an initial increase followed by a decrease in thermal stability is also attributed to the agglomeration of the two-dimensional layers within the epoxy matrix at higher filler content.

**Figure 8:**
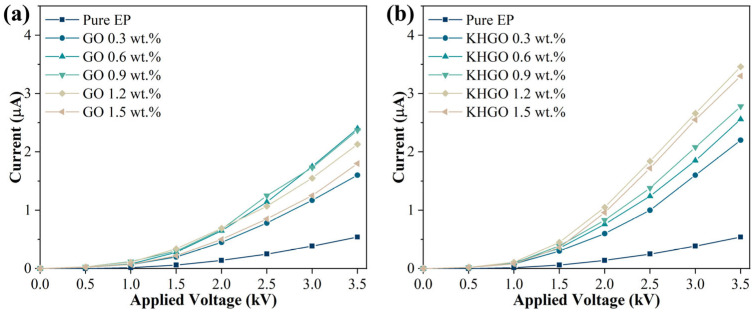
Current through GO/EP composites (**a**) and KHGO/EP composites (**b**) as a function of applied voltage.

An external DC voltage was applied to GO/EP composites and KHGO/EP composites with varying filler content, and the current passing through the samples was measured, as shown in Figure 8. It can be observed that as the applied voltage increases, the current through the samples also increases; however, the current–voltage relationship is nonlinear. This nonlinear behavior can be explained using the trap theory of polymer composites. The presence of numerous traps within the polymer composites hinders the movement of charge carriers, thereby reducing the bulk conductivity of the material. As the external voltage increases, these traps gradually become filled, diminishing their obstructive effect on the movement of charge carriers. Consequently, the bulk conductivity increases with increasing external voltage, resulting in the current–voltage characteristics shown in Figure 8.

**Figure 9:**
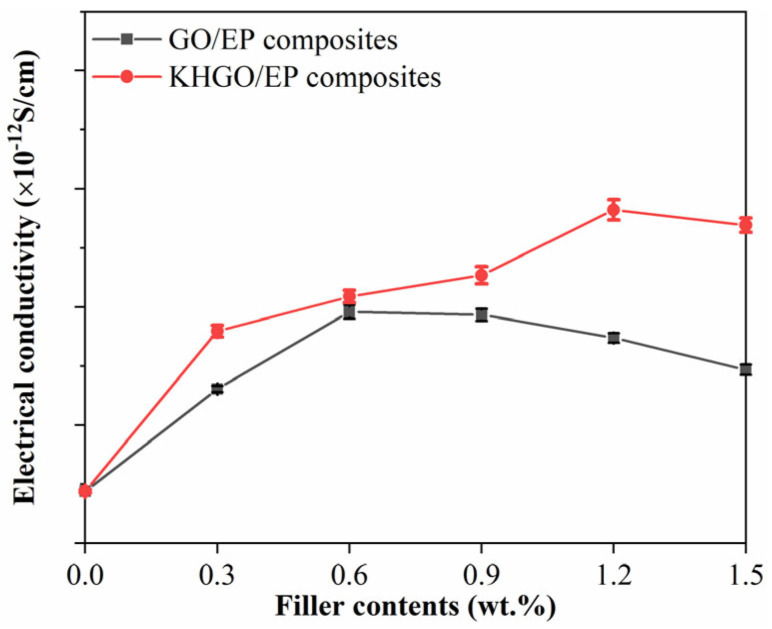
Volume electrical conductivity of GO/EP composites and KHGO/EP composites with different filler content.

The volume electrical conductivity of composite samples with different GO and KHGO content under an external DC voltage of 3.5 kV is shown in Figure 9. The results indicate that, with the increase in filler content, both GO/EP composites and KHGO/EP composites exhibit an initial increase followed by a decrease in volume electrical conductivity. The volume electrical conductivity of pure epoxy resin is approximately 5 × 10^−12^ S/cm^2^. When the GO content increases to 0.6 wt.%, it reaches a maximum value of about 20 × 10^−12^ S/cm^2^; when the KHGO content increases to 1.2 wt.%, it reaches a maximum value of about 30 × 10^−12^ S/cm^2^. It is evident that the variation pattern of the volume electrical conductivity of composite samples with different filler content is similar to the variation pattern of thermal conductivity with filler content. This is because, compared to pure epoxy resin, the factors that cause changes in the volume electrical conductivity of composites are mainly twofold: first, the electrical conductivity of the inorganic filler itself is higher than that of the polymer matrix; second, the interface between the inorganic filler and the polymer matrix often becomes a channel for charge transfer. The reason for the decrease in volume electrical conductivity with increasing filler content beyond the critical value, similar to the change in thermal conductivity, is due to the aggregation of the 2D sheets within the polymer matrix when the filler content exceeds the critical value.

### 3.3. PALS Spectra of GO/EP Composites and KHGO/EP Composites

**Figure 10:**
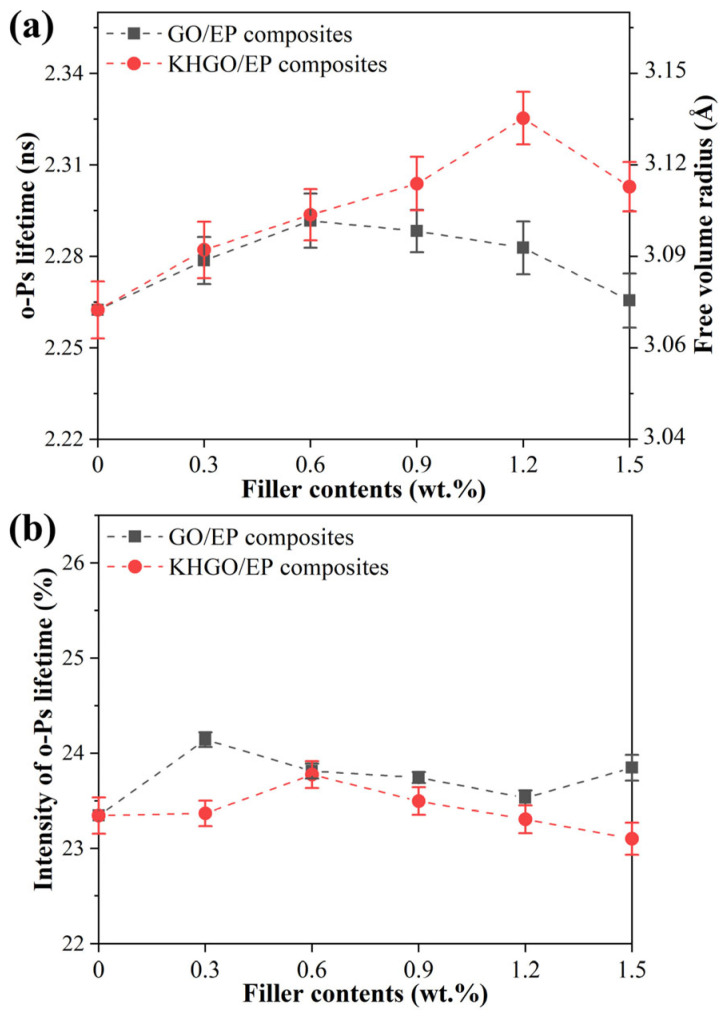
The *o*-Ps lifetime and average size of the free-volume holes of GO/EP composites and KHGO/EP composites (**a**); intensity of *o*-Ps lifetime (**b**).

As shown in Figure 10, the positron annihilation lifetime spectra of GO/EP and KHGO/EP composites with different filler content are presented. Figure 10a shows the *o*-Ps annihilation lifetime with the average dimension of free volume holes calculated by *o*-Ps lifetime [50,51]. Figure 10b shows the *o*-Ps annihilation intensities. From Figure 10a, it can be observed that with increasing GO content, the *o*-Ps annihilation lifetime in GO/EP composites increases. However, when the GO content exceeds 0.6 wt.%, the *o*-Ps annihilation lifetime decreases with further increases in GO content. Similarly, the *o*-Ps annihilation lifetime in KHGO/EP composites increases with increasing KHGO content but decreases when the KHGO content exceeds 1.2 wt.%. Figure 10b indicates that the *o*-Ps annihilation intensity remains relatively constant with varying filler content in both GO/EP and KHGO/EP composites.

The positron annihilation theory in polymer materials suggests that the *o*-Ps annihilation lifetime is related to the average size of free-volume holes in the polymer. The larger the average size of these free volume holes, the longer the *o*-Ps annihilation lifetime. Free volume in polymers is the unoccupied space arising from the free rotation of polymer chains and the presence of terminal groups and side chains uniformly distributed throughout the polymer matrix on a larger scale. When inorganic fillers are added to the polymer, compatibility issues between the organic matrix and inorganic fillers create defects at the filler-matrix interface. These defects are theoretically larger than the intrinsic free-volume holes in the polymer. As the GO or KHGO content increases, the total volume of interfaces between the layers and the polymer matrix increases, leading to a longer *o*-Ps annihilation lifetime. When the GO or KHGO content exceeds a critical value, the layers aggregate within the matrix due to van der Waals forces, reducing the total volume of the interface layer and subsequently decreasing the *o*-Ps annihilation lifetime. Furthermore, at the same content, the *o*-Ps average lifetime in KHGO/EP composites is slightly longer than that in GO/EP composites, and the critical content is higher. This is because KHGO has been modified with silane coupling agents, introducing organic groups, particularly epoxy groups, which enhance its dispersion in the epoxy matrix according to the principle of similar compatibility.

### 3.4. EIS Spectra of KHGO/EP Composites after Corona Discharge Treatment

Figure S6 show the EIS diagrams (Bode plots) of pure epoxy resin without corona treatment after immersion in NaCl solution (3.5 wt.%) for different times and the corresponding equivalent circuits. The EIS spectra at different immersion times can be fitted using the equivalent circuit, as shown in Figure S6, where *R*_e_ resistance of the NaCl solution, *R*_b_ represents the resistance of the epoxy resin sample, and CPE_b_ is the constant phase element representing the sample capacitance *C*_b_. In EIS tests, the actual sample capacitance is not ideal due to surface imperfections and defects. Consequently, this study employs a constant phase element (CPE) to model the capacitance. The capacitance value *C*_b_ of the sample can be calculated from the CPE value obtained from the equivalent circuit fitting [52]. The EIS diagrams (Bode plots) and equivalent circuits of pure epoxy resin treated with 6 kV corona discharge for 3 h are shown in Figure S7. In the initial immersion period of the sample, there were two time constants presented. The high-frequency time constant corresponds to the sample’s capacitance, while the low-frequency time constant generally reflects the double-layer capacitance that forms at the interface between the sample and the ITO glass as a result of aqueous solution diffusion. When the immersion time reached 72 h, two new time constants appeared at intermediate frequencies, indicating the presence of defects at different depths within the sample due to corona corrosion.

**Figure 11:**
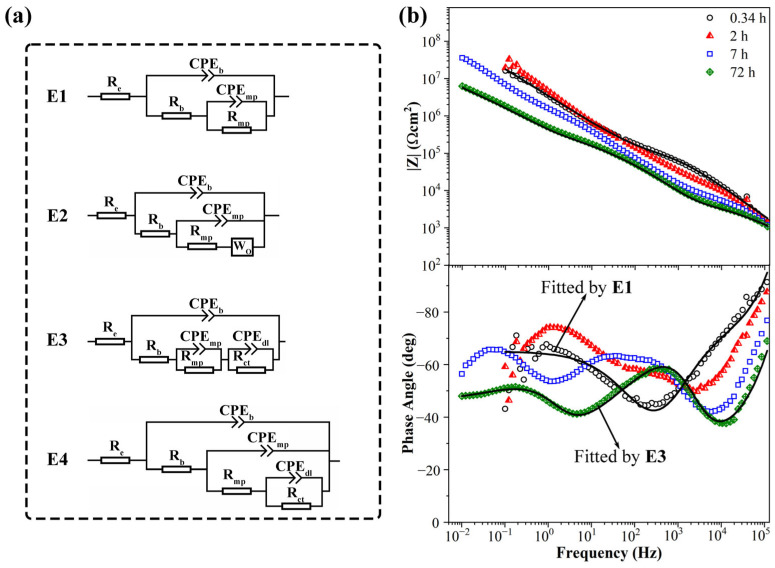
Equivalent circuits in this article (**a**); impedance diagrams (Bode plots) of 0.3 wt.% KHGO/EP composites treated with corona discharge after immersion for different times (**b**).

The equivalent circuits of EIS diagrams used in this article are shown in Figure 11a. The EIS diagrams of 0.3 wt.% KHGO/EP composites treated with 6 kV corona discharge for 3 h after immersion in 3.5 wt.% NaCl solution are shown in Figure 11b. At the initial stage of immersion, when the immersion time reached 0.34 h, the Bode plots revealed two time constants. The time constant between 10^5^ Hz and 10^2^ Hz corresponds to the sample capacitance (*C*_b_) and the sample resistance (*R*_b_). The time constant between 10^2^ Hz and 10^−1^ Hz is more peculiar; at low frequencies, the phase angle gradually increases as the frequency decreases, exhibiting characteristics of Warburg impedance, which represents the diffusion process of the aqueous solution. However, near 10^−1^ Hz, the phase angle-frequency curve bends downward, leading to the use of a CPE element to fit the low-frequency time constant, as shown in equivalent circuit E1. As the immersion time increased, the phase angle at high frequencies decreased, indicating that the aqueous solution diffused into the sample, resulting in increased sample capacitance and decreased resistance. By the time the immersion reached 2 h, the Bode plots of the sample showed three time constants: the high frequency (10^5^ Hz to 10^3^ Hz) time constant represents the sample’s capacitance and resistance; the middle frequency (10^3^ Hz to 10^2^ Hz) time constant corresponds to the micropore capacitance (CPE_mp_) and micropore resistance (*R*_mp_) caused by corona-induced defects within the sample; and the low-frequency time constant represents the double-layer capacitance (CPE_dl_) and charge transfer resistance (*R*_ct_) at the interface between the sample and the ITO glass due to the diffusion of the aqueous solution. As the immersion time extended further, the phase angle at high frequencies continued to decline, and the impedance modulus at low frequencies decreased from nearly 10^8^ Ω·cm^2^ to below 10^7^ Ω·cm^2^, indicating that the sample had absorbed an amount of aqueous solution. The impedance spectrum at 72 h of immersion can be fitted using either equivalent circuit E3 or E4, but E3 provides a better fit. This equivalent circuit suggests that a substantial amount of the aqueous solution accumulated at the interface between the sample and the ITO glass, resulting in a larger area of the solution layer.

**Figure 12:**
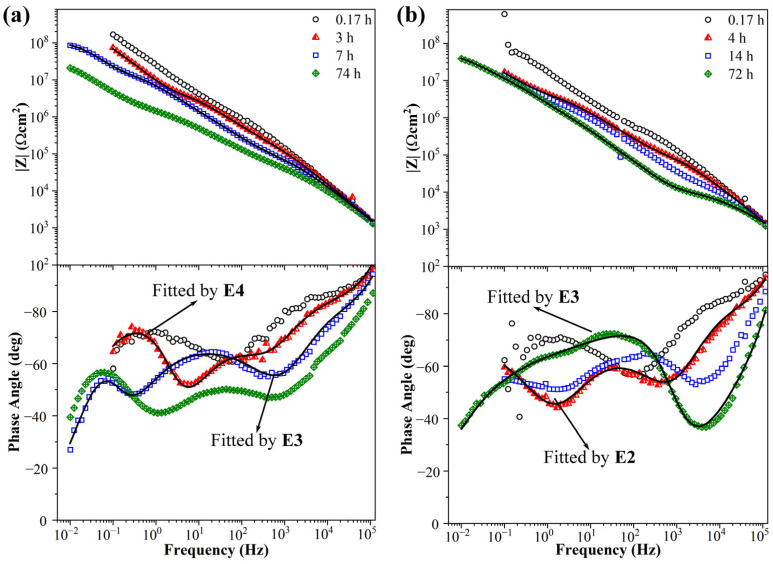
Impedance diagrams (Bode plots) of 0.6 wt.% (**a**) and 0.9 wt.% (**b**) KHGO/EP composites treated with corona discharge after immersion for different time.

The impedance spectra of the 0.6 wt.% KHGO/EP composite sample treated with corona discharge after immersion in NaCl solution for different times are shown in Figure 12a. Similar to the 0.3 wt.% sample, the Bode plots of this sample initially exhibit two time constants. However, overall, the 0.6 wt.% sample’s impedance spectrum displays a higher phase angle at high frequencies and a larger impedance modulus at low frequencies. After 3 h of immersion, a third time constant emerges in the middle-frequency range (10^3^ Hz to 10^1^ Hz), which can be fitted using equivalent circuit E4. From high to low frequency, the three time constants correspond to the sample capacitance, the micropore capacitance caused by defects, and the double-layer capacitance resulting from the accumulation of the aqueous solution at the interface. Based on the previous discussion, it can be concluded that after 3 h of immersion, the aqueous solution has already diffused into the defects created by corona treatment and has passed through these defects to reach the interface between the sample and the ITO glass. As the immersion time increases, the phase angle at high frequencies significantly decreases, and the impedance modulus at low frequencies drops to around 10^7^ Ωcm^2^. By the time the immersion reaches 7 h, the impedance spectrum of this sample can be fitted using equivalent circuit E3, indicating that during prolonged immersion, the aqueous solution has permeated through the sample and accumulated at the ITO glass surface, forming a relatively significant solution layer. Simultaneously, the time constants at middle frequencies become more distinct from those at high frequencies, suggesting that the aqueous solution continues to diffuse into the sample and fills the defects created by the corona treatment. Additionally, the impedance spectra at different immersion times reveal that, during the initial immersion, there is an overlap between the capacitive arcs representing the sample capacitance and the micropore capacitance caused by defects. This overlap is due to the limited water absorption in the early stages, which results in a higher phase angle at high frequencies, and the aqueous solution has not yet fully filled the corona-induced defects.

The Bode plots of EIS spectra for the 0.9 wt.% KHGO/EP composite sample after corona treatment and immersion in NaCl solution are shown in Figure 12b. In the initial stages of immersion, the Bode plot of this sample closely resembles those of the previous two samples, with a second time constant appearing at low frequencies, exhibiting the characteristics of Warburg impedance. Additionally, the sample demonstrates a high phase angle at high frequencies and a high impedance modulus at low frequencies. As the immersion time increases, the phase angle at high frequencies decreases, and the impedance modulus at low frequencies also decreases. This is attributed to the increase in capacitance and the decrease in resistance caused by the water absorption process. The Bode plots after 4 h of immersion can be fitted using equivalent circuit E2, where the time constant at high frequencies represents the sample capacitance, the time constant at middle frequencies represents the micropore capacitance and micropore resistance due to defects generated by the corona treatment, and the time constant at low frequencies, characterized by Warburg impedance, represents the diffusion process of the aqueous solution. After 72 h of immersion, the Bode plots can be fitted using equivalent circuit E3, indicating that the aqueous solution has diffused to the interface between the sample and the ITO glass, forming an accumulated solution layer. There is an overlap between the time constant representing micropore capacitance and the time constant representing the double-layer capacitance formed by the diffusion of the aqueous solution to the interface. This overlap might be due to the relatively small amount of aqueous solution that has diffused to the interface, causing the characteristics of the double-layer capacitance to be masked by those of the micropore capacitance. The impedance modulus at low frequencies after 72 h of immersion is close to 10^8^ Ωcm^2^, indicating that the sample has absorbed a relatively small amount of water.

**Figure 13:**
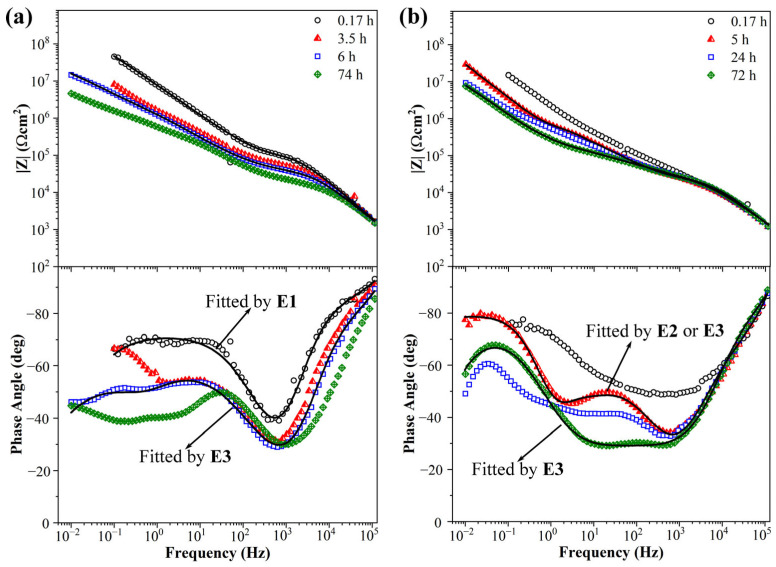
Impedance diagrams (Bode plots) of 1.2 wt.% (**a**) and 1.5 wt.% (**b**) KHGO/EP composites treated with corona discharge after immersion for different times.

The impedance spectra of the 1.2 wt.% KHGO/EP composite samples treated with corona discharge after immersion at different times are shown in Figure 13a. In the initial immersion period, the Bode plots exhibit a noticeable decrease in phase angle at high frequencies compared to the previous three samples, while maintaining a relatively high impedance modulus at low frequencies. This result indicates a faster diffusion rate of the solution at the beginning of immersion. After 0.17 h of immersion, the Bode plots show two time constants, which can be fitted using the equivalent circuit E1. It is worth noting that although the low-frequency time constant can be fitted with a single CPE element, the curve shape suggests that a third time constant, representing the diffusion of the solution to the interface between the sample and the ITO glass, has already begun to emerge. After 3.5 h of immersion, a third time constant appears in the impedance spectra, with the low-frequency time constant exhibiting characteristics of Warburg impedance, reflecting the diffusion process of the solution. After 6 h of immersion, the Warburg impedance characteristics disappear, and the impedance spectrum can be fitted using the equivalent circuit E3, with the impedance modulus at low frequencies decreasing to around 10^7^ Ωcm^2^. As the immersion time extends to 72 h, the sample continues to show a significant decrease in the high-frequency phase angle, while the low-frequency impedance spectrum falls below 10^7^ Ωcm^2^, indicating a relatively high saturation water absorption level in the sample.

The impedance spectra of the KHGO/EP composite with a filler content of 1.5 wt.% after corona treatment, immersed in NaCl solution, are shown in Figure 13b. The impedance spectra at different immersion times reveal a distinct characteristic: at the initial period of immersion, the sample shows a low phase angle at high frequencies, and as the immersion time increases, the high-frequency impedance spectra nearly overlap. This result indicates that the sample absorbs water very quickly, reaching near saturation in the initial immersion stage. The impedance spectra show two time constants during the initial immersion period. However, fitting the equivalent circuit revealed that the impedance spectrum at 0.17 h could not be fitted using E1, which is different from the four samples with lower KHGO content. This discrepancy arises because the higher KHGO content in this sample causes more extensive exposure of KHGO when the epoxy resin matrix degrades under corona treatment. This exposure leads to the formation of complex surface and internal defect structures, making it challenging to fit the impedance spectrum with a simple model. After 5 h of immersion, a third time constant appears in the impedance spectrum, and the distinction between the two time constants at the intermediate and low frequencies becomes more pronounced, indicating that a significant amount of the solution has diffused to the interface between the sample and the ITO glass. As the immersion time extends, the impedance spectra at 24 h and 72 h both exhibit three time constants, which can be fitted using the equivalent circuit E3. This result further demonstrates that the solution diffusion rate in this sample is rapid, leading to a near-saturation state of water absorption within a relatively short period.

**Figure 14:**
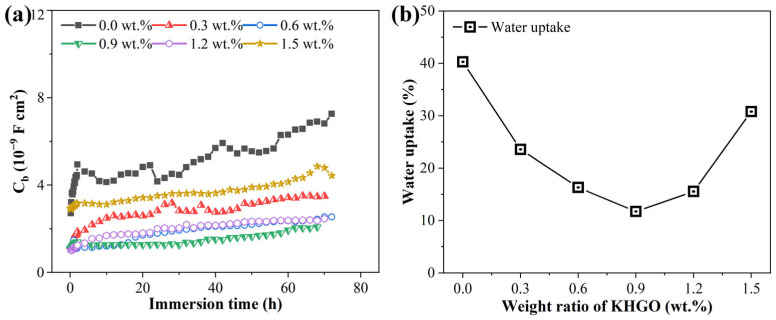
Variation of *C*_b_ of corona-treated KHGO/EP composites with different filler content as a function of immersion time (**a**); water uptake of KHGO/EP composites after immersion for 72 h as a function of filler content (**b**).

The variation of the capacitance *C*_b_ with immersion time for epoxy resin composite samples containing different KHGO content after 3 h of corona treatment at 6 kV and subsequent immersion in NaCl solution was determined using equivalent circuit fitting. The results are shown in Figure 14a. The *C*_b_ of all samples demonstrates a comparable trend over immersion time: the capacitance initially rises sharply, followed by a gradual slowdown in the rate of increase. The magnitude of *C*_b_ is related to the amount of water absorbed by the sample, meaning the faster the diffusion of the solution, the faster the change in *C*_b_. This trend arises because the diffusion rate is correlated with the concentration difference—the larger the concentration difference, the faster the diffusion. Figure 14a also reveals that as the KHGO content increases, the overall *C*_b_ decreases. However, when KHGO content exceeds 0.9 wt.%, the overall *C*_b_ increases with increasing filler content, with the *C*_b_ of the 1.5 wt.% KHGO sample approaching that of pure epoxy resin. The water uptake rate of the samples after 72 h of immersion can be calculated by the value of *C*_b_ [53,54] and the results are shown in Figure 14b. As shown in the figure, the water absorption rate decreases as the KHGO content increases; however, when the KHGO content exceeds 0.9 wt.%, the water absorption rate increases with further increases in KHGO content. The results of the AC impedance tests and analysis indicate that the composite sample with 0.9 wt.% KHGO has the lowest water absorption rate.

**Figure 15:**
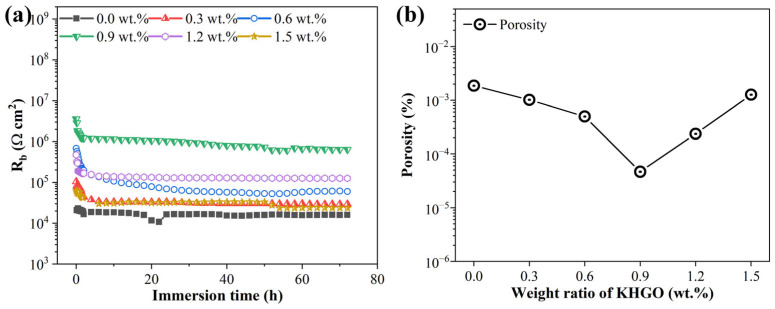
Variation of *R*_b_ of corona-treated KHGO/EP composites with different filler content as a function of immersion time (**a**); porosity of KHGO/EP composites after immersion for 72 h as a function of filler content (**b**).

Using the equivalent circuit method, the variation of the resistance *R*_b_ with immersion time for samples with different KHGO content, after corona treatment and subsequent immersion in NaCl solution was calculated. The results are shown in Figure 15a. As the KHGO content increases, the overall *R*_b_ of the samples increases during immersion. However, once the KHGO content surpasses 0.9 wt.%, the overall *R*_b_ decreases with increasing KHGO content. The figure also shows that after 40 h of immersion, the resistance values obtained from fitting become stable over time. The porosity was calculated based on the *R*_b_ values obtained after 72 h of immersion [55]. The results are shown in Figure 15b. With an increase in filler content, the porosity of the samples first decreases and subsequently increases, with the composite sample containing 0.9 wt.% KHGO exhibiting the lowest porosity.

**Figure 16:**
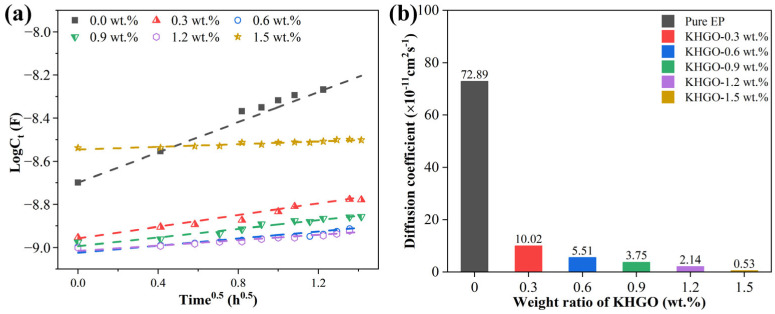
Log(*C*_t_) as a function of h^0.5^ of KHGO/EP composites with different filler content at the initial immersion period (**a**); diffusion coefficient as a function of filler content (**b**).

Assuming that the diffusion process of the solution into the samples during the initial immersion phase follows Fick’s diffusion law, the square root of the diffusion coefficient *D* (denoted as *D*^0.5^) should exhibit a positive linear relationship with the ratio of the logarithm of the capacitance to the square root of the diffusion time log*C_t_*/*t*^0.5^. To investigate this, a plot of the logarithm of the capacitance log*C_t_* against the square root of the immersion time *t*^0.5^ was created for epoxy resin composite samples with different KHGO content that had undergone corona treatment, as shown in Figure 16a. The data were then linearly fitted [56]. From the figure, it can be observed that the slope of the fitted line for the pure epoxy resin sample is the largest, while the slope of the fitted line for samples with higher filler content is smaller. By converting the slopes of the fitted lines, the diffusion coefficients for the corona-treated epoxy composite samples with different KHGO content during the initial immersion phase were calculated, as shown in Figure 16b. As the KHGO content increases, a continuous decrease in the diffusion coefficient is observed.

The changes in electrochemical impedance spectroscopy (EIS) over time essentially reflect the diffusion process of the aqueous solution within the sample, which is determined by the sample’s microstructure. Thus, the impedance spectra, equivalent circuits, and the parameters obtained from fitting, such as porosity and diffusion coefficient, effectively represent the structural degradation of the epoxy resin composites after corona treatment. The experimental and fitting results of this study indicate that, under identical corona discharge conditions, the epoxy resin samples containing KHGO exhibit fewer time constants, lower saturation water absorption, and reduced porosity compared to pure epoxy resin samples. This suggests that the addition of KHGO enhances the corona resistance of the epoxy resin. With the increase in KHGO content, the saturation water absorption and porosity of the epoxy resin composites after corona treatment first increase and then decrease. Notably, this trend is consistent with the observed variations in thermal conductivity, thermal stability, and volume conductivity of the composites. Based on the positron annihilation lifetime spectroscopy (PALS) results, it is inferred that this initial increase followed by a decrease is due to the dispersion and agglomeration of the two-dimensional nanosheets within the epoxy matrix. Additionally, the EIS results demonstrate that the diffusion coefficient of the aqueous solution in the initial stage of immersion decreases continuously with increasing KHGO content. When the KHGO content reaches 1.5 wt.%, the diffusion coefficient in the early stage of immersion is as low as 0.53 × 10^−11^ cm^2^s^−1^, likely due to the severe aging of the sample and the agglomerated fillers that actually facilitate the diffusion of the aqueous solution. This rapid diffusion process results in a high initial *C*t value with minimal change over time, as shown in Figure 16a. This explains the extremely low diffusion coefficient calculated from the formula. Combining the analysis of equivalent circuits, saturation water absorption, porosity, and diffusion coefficients, it can be concluded that even a small amount of uniformly dispersed KHGO nanosheets can effectively improve the corona resistance of epoxy resin composites. The likely mechanism behind this enhanced corona resistance is that the high aspect ratio of KHGO nanosheets effectively blocks the high-energy particles generated by corona discharge while also improving the thermal conductivity and thermal stability of the epoxy matrix, thereby mitigating the local overheating and aging caused by corona discharge.

## 4. Conclusions

In summary, functionalized graphene oxide (KHGO) was successfully synthesized, and the corona resistance of KHGO/EP composites was thoroughly investigated. After corona treatment, the saturation water uptake and porosity of the KHGO/EP composites initially decreased but increased when the KHGO content exceeded 0.9 wt.%. EIS results showed that the 0.9 wt.% KHGO/EP composite exhibited the fewest internal defects and minimal structural damage, demonstrating the highest corona resistance. PALS analysis revealed that KHGO disperses well within the epoxy matrix at lower concentrations but tends to aggregate beyond a certain threshold. Furthermore, the thermal properties of KHGO/EP composites suggest that well-dispersed KHGO significantly enhances both the thermal conductivity and stability of the epoxy resin. The improved corona resistance is likely due to the physical barrier effect of the well-dispersed KHGO and its capacity to enhance the resin’s thermal characteristics. However, aggregation induced by van der Waals forces remains a key factor limiting further improvements in corona resistance. This study demonstrates that the incorporation of two-dimensional sheet-like fillers offers a promising approach to enhancing the corona resistance of polymer insulation materials. Enhancing the dispersion of these fillers within the polymer matrix presents a reliable and promising direction for future research.

## Data Availability

Raw data is available upon request.

## Supplementary Materials

The following supporting information can be downloaded at: https://www.mdpi.com/article/10.3390/ma17194864/s1, Figure S1: Schematic diagram of surface modification mechanism of GO; Figure S2: Schematic diagram of corona discharge apparatus; Figure S3: XPS survey spectra of GO and KHGO; Figure S4: SEM image (a) and TEM (b) image of GO; elemental distribution on the surface of GO (c–f); Figure S5: TG curves of GO/EP composites (a) and KHGO/EP composites (b) with different filler content from 30 °C to 600 °C; Figure S6: EIS diagrams and equivalent circuits of pure epoxy resin after immersion for different time; Figure S7: EIS diagrams and equivalent circuits of pure epoxy resin treated with 6 kV corona discharge for 3 h after immersion for different time.
