# Supplementary material for: Significantly Enhanced Corona Resistance of Epoxy Composite by Incorporation with Functionalized Graphene Oxide
Source: Materials (Basel). 2024 Oct 2;17(19):4864. doi: 10.3390/ma17194864 (PMC11478297; doi:10.3390/ma17194864)
Supplement: Supplementary file 1 [file materials-17-04864-s001.zip › materials-3217382-supplementary.pdf]

Supporting Information

Significantly Enhanced Corona Resistance of Epoxy Composites Reinforced with Epoxy-Functionalized Graphene Oxide Nanosheets

Yue Yang[1], Yumin Wang[1], Chunqing He[1], Zheng Wang [2], Xiangyang Peng [2], Pengfei Fang[1,*]

1 School of Physics and Technology, Wuhan University, Wuhan 430072, China; yangyue@whu.edu.cn (Y.Y.); 1224839088@qq.com (Y.W.); hecq@whu.edu.cn (C.H.)

2 Guangdong Key Laboratory of Electric Power Equipment Reliability, Electric Power Research Institute of Guangdong Power Grid Co., Ltd, Guangzhou 510080, China; williamatwhu@163.com (Z.W.); pigpxy@126.com (X.P.)

* Correspondence: fangpf@whu.edu.cn (P.F.)

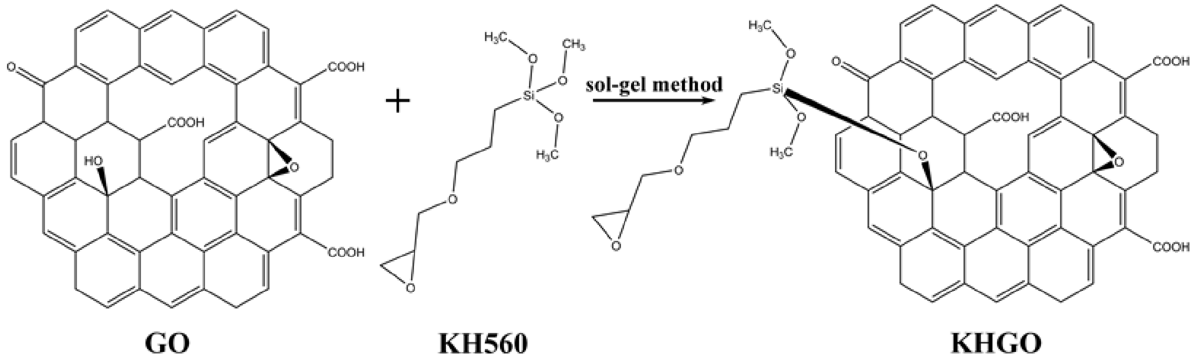

Figure S1. Schematic diagram of surface modification mechanism of graphene oxide

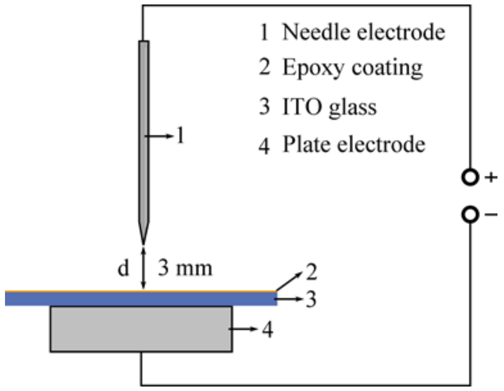

Figure S2. Schematic diagram of corona discharge apparatus

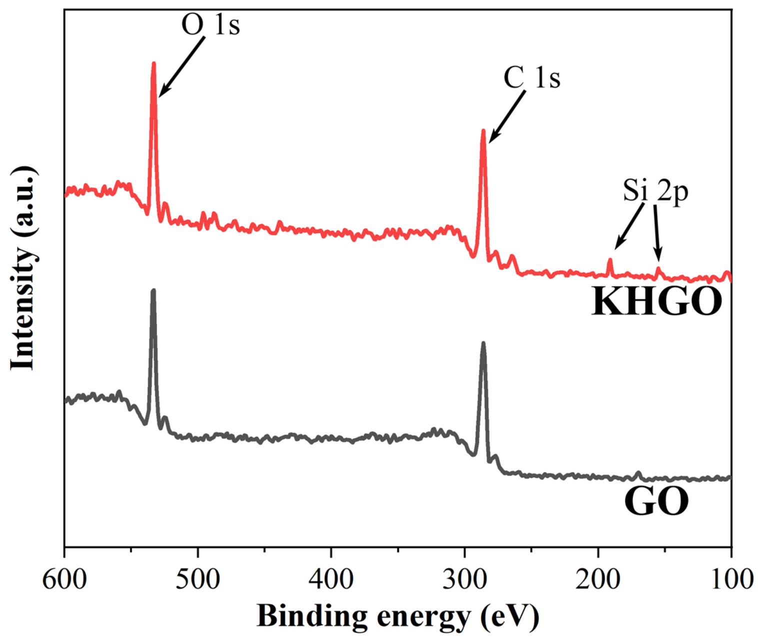

Figure S3. XPS survey spectra of GO and KHGO

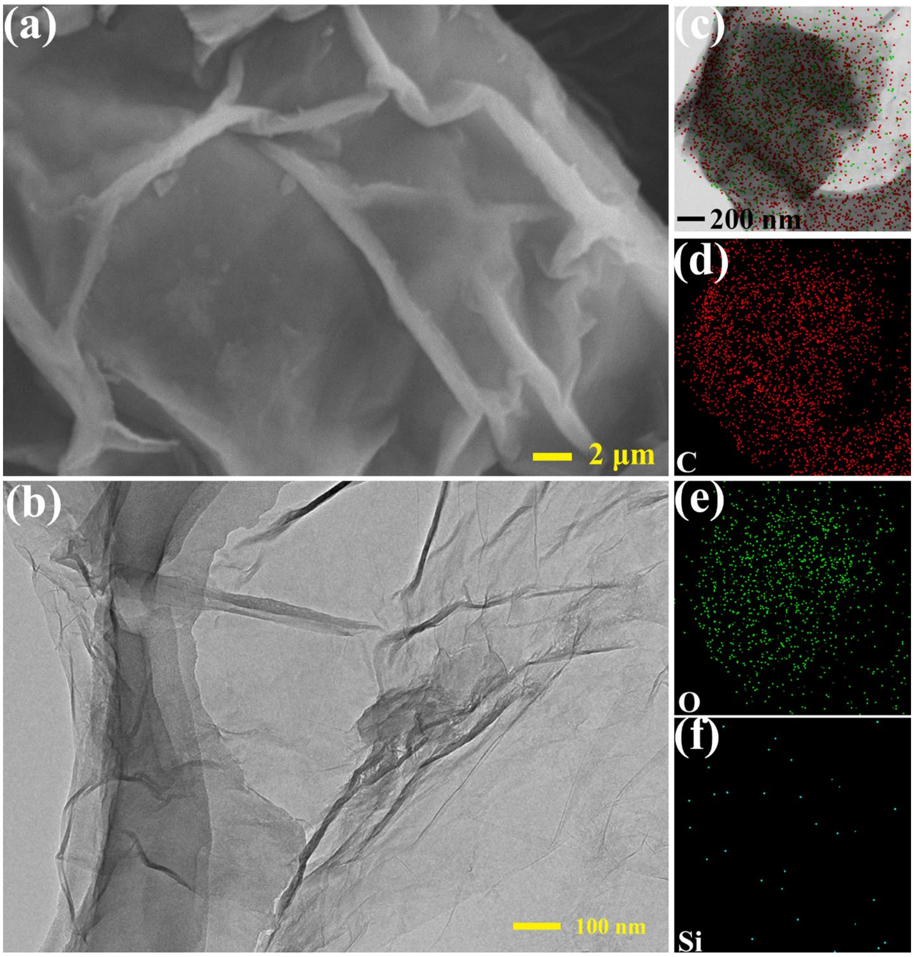

Figure S4. SEM image (a) and TEM image (b) of GO; elemental distribution on the surface of GO (c-f)

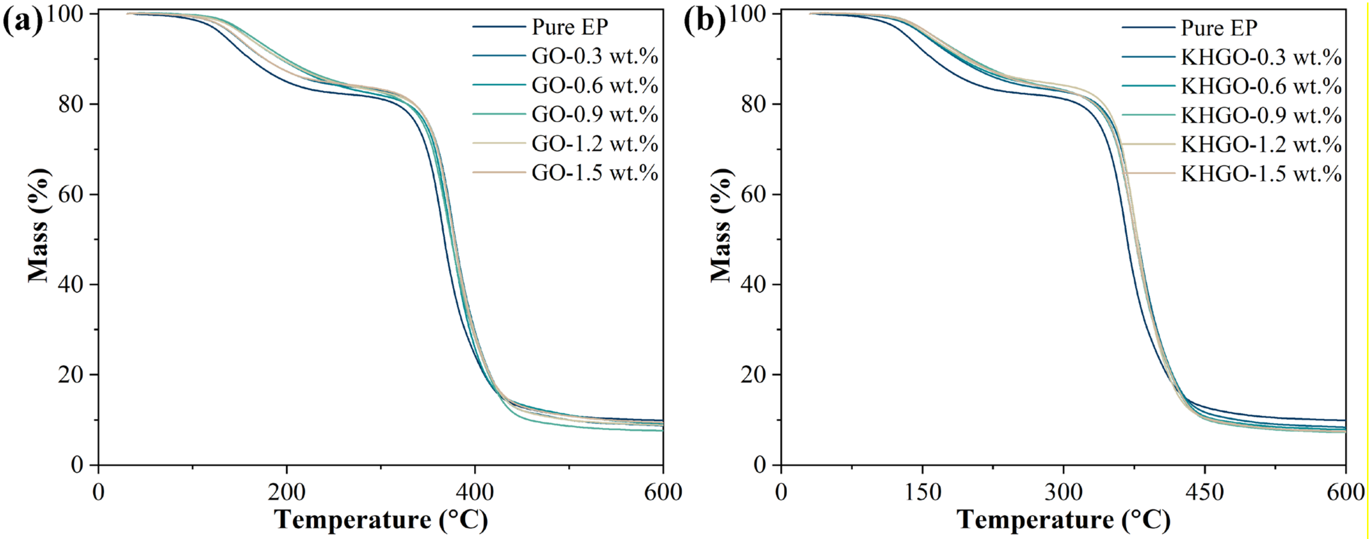

Figure S5. TG curves of GO/EP composites (a) and KHGO/EP composites (b) with different filler contents from 30 °C to 600 °C

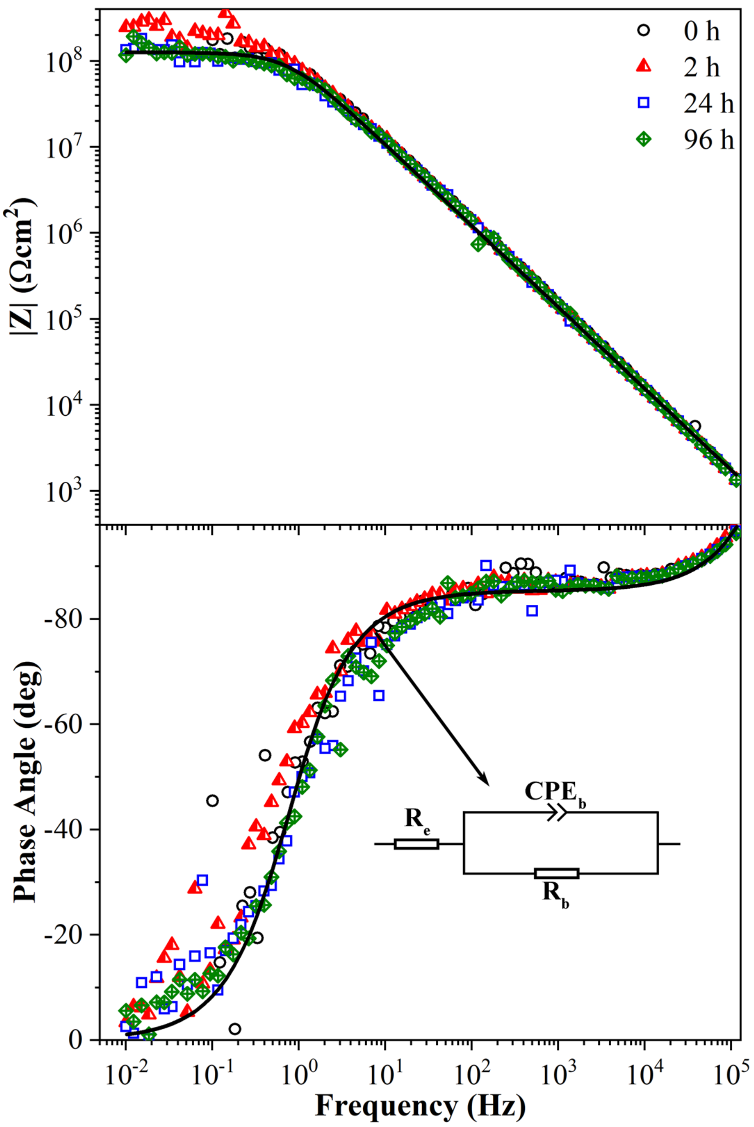

Figure S6. EIS diagrams and equivalent circuits of pure epoxy resin after immersion for different time

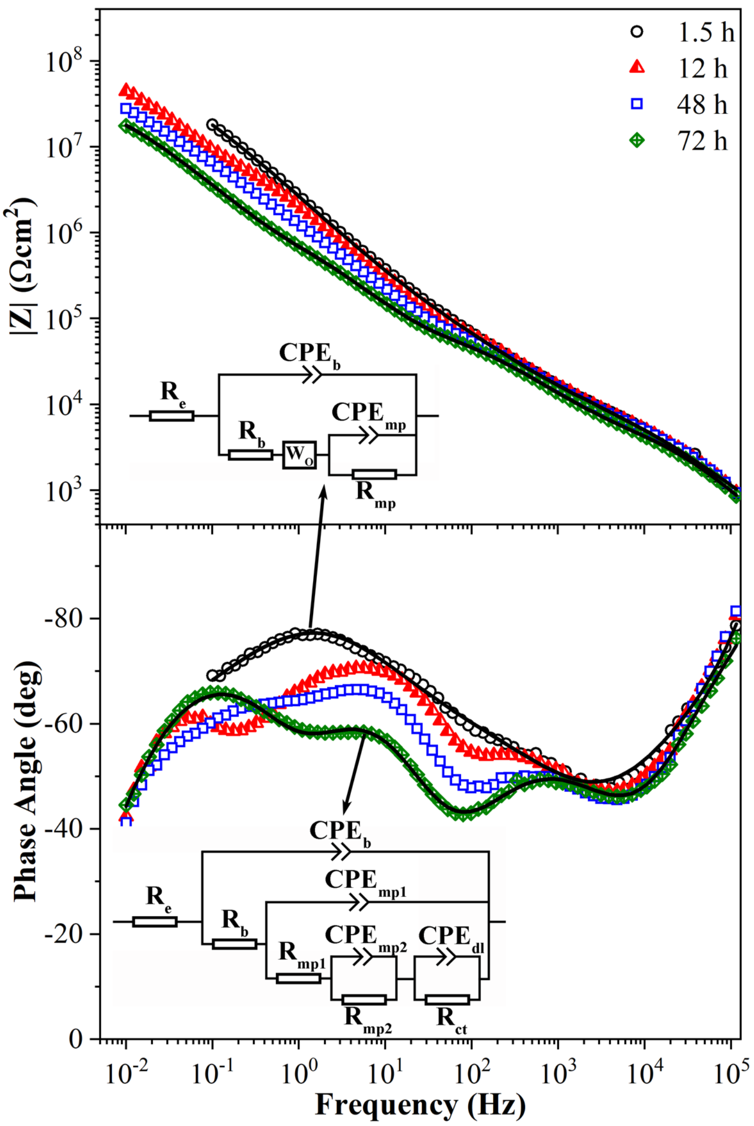

Figure S7. EIS diagrams and equivalent circuits of pure epoxy resin treated with 6 kV corona discharge for 3 h after immersion for different time
